# Chinese herbal medicine (Ma Zi Ren Wan) for functional constipation: study protocol for a prospective, double-blinded, double-dummy, randomized controlled trial

**DOI:** 10.1186/1745-6215-14-366

**Published:** 2013-11-04

**Authors:** Linda LD Zhong, Chung Wah Cheng, Yawen Chan, King Hong Chan, Ting Wa Lam, Xiao Rui Chen, Chi Tak Wong, Justin CY Wu, Zhao Xiang Bian

**Affiliations:** 1 School of Chinese Medicine, Hong Kong Baptist University, 1/F, Jockey Club School of Chinese Medicine Building, 7 Baptist University Road, Kowloon Tong, Hong Kong, SAR, China; 2 Institute of Digestive Disease, The Chinese University of Hong Kong, Room 94020, 7/F Clinical Sciences Building, Prince of Wales Hospital, Shatin, NT, Hong Kong, SAR, China; 3 Department of Family Medicine & General Out-patient Clinics, KCC Cluster, Hospital Authority, 30 Gascoigne Road, Kowloon, Hong Kong, SAR, China; 4 Department of Medicine, Queen Elizabeth Hospital, Hospital Authority, 30 Gascoigne Road, Kowloon, Hong Kong, SAR, China

**Keywords:** Functional constipation, Chinese herbal medicine, Ma Zi Ren Wan, Randomized controlled trial

## Abstract

**Background:**

Functional constipation is a common clinical complaint. Although the effectiveness of Ma Zi Ren Wan for alleviating functional constipation symptoms has been proven in a previous randomized placebo-controlled study, further evidence is needed to make clinical recommendations about Chinese herbal medicine. In particular, a comparison with conventional western medicine for functional constipation patients is needed.

**Methods/Design:**

This is a prospective, double-blinded, double dummy, randomized, controlled trial. After a 2-week run-in period, eligible patients (Rome III) with excessive traditional Chinese medicine syndrome will randomly be assigned to the Chinese medicine arm (Ma Zi Ren Wan and western medicine placebo), western medicine arm (senna and Chinese medicine placebo) or placebo arm (Chinese medicine placebo and western medicine placebo). Patients will undergo an 8-week treatment and an 8-week follow-up. The primary outcome is the responder rate for complete spontaneous bowel movement (CSBM) during treatment. Patients with a mean increase of CSBM ≧1/week in comparison with their baselines are defined as responders. The secondary outcomes include responder rate during follow-up, changes of colonic transit as measured with radio-opaque markers, individual and global symptom assessments, and reported adverse effects.

**Discussion:**

This study is the first study to compare a Chinese Herbal Medicine (Ma Zi Ren Wan) with a laxative that is commonly used in the clinical practice of western medicine, and with a placebo. This study will complete the investigation of Ma Zi Ren Wan for functional constipation, and should, therefore, suggest recommendations for clinical practice. Furthermore, the process of first conducting a systematic review, then implementing a dose determination study followed by a placebo-control trial, and finally, comparing traditional Chinese medicine with an active conventional medicine in a controlled trial can be a reference to other researches on Chinese medicine interventions in the future.

**Trial registration:**

NCT01695850

## Background

Constipation affects a substantial portion of the population, involving an estimated 12% to 19% of Americans [1], 14% of Asians [2], and up to 27% of the global population, depending on demographic factors, sampling, and definition [3]. Treatments for constipation usually include fiber supplements, osmotic and stimulant laxatives, stool softeners, and sometimes enemas for refractory constipation [4]. With the unsatisfactory response to current symptomatic treatments [5], many patients seek help from traditional Chinese medicine (TCM), mostly by taking Chinese herbal medicine (CHM) [4].

According to TCM theory, constipation can be divided into excessive and deficient patterns based on the underlying etiology [6-8]. The former is characterized by the presence of Heat or pathological accumulation of Qi (Qi stagnation), while the latter is characterized by the deficiency of Qi, Ying or Yang. The patent formula Ma Zi Ren Wan (MZRW) was first recorded in the TCM classic, 'Discussion of Cold-induced Disorders’ (ShangHanLun) [6], and this formula has been commonly used for excessive constipation in China and other Asian countries for more than two thousand years since the Han Dynasty (A.D. 200). It comprises six herbs: *Semen Cannabis Sativae*, *Semen Pruni Armeniacae*, *Radix Paeoniae*, *Fructus Immaturus Citri Aurantii*, *Cortex Magnoliae* and *Radix et Rhizoma Rhei*. Through the combined action of these herbs, MZRW can moisten the intestines, drain Heat, promote the movement of Qi and unblock the bowel [9]. Based on modern pharmaceutical studies, MZRW can stimulate intestinal mucosa, increase secretion, accelerate intestinal peristalsis and decrease water absorption [10,11].

Our research team began studying MZRW for functional constipation (FC) in 2006. We found that available studies had significant methodological flaws and lacked replicable validation; thus, clinical findings could not be definitively determined [12]. Therefore, the efficacy and safety of MZRW had to be justified by first determining the optimal dose. The currently recommended dose (5.0 g b.i.d.) was compared with higher (7.5 g b.i.d.) and lower doses (2.5 g b.i.d.); the study was supported by the WooFo Medical Foundation. The dose of 7.5 g b.i.d. was found to have better therapeutic effect than that of 2.5 and 5.0 g b.i.d. among 96 subjects (32 per arm). Second, MZRW in optimal dosage (7.5 g b.i.d.) was compared with a placebo for FC patients with excessive TCM Syndrome in a randomized, double-blind clinical trial, which was supported by the Health and Health Services Research Fund. In this study, 120 subjects were randomized into two arms (60 per arm). Response rates for the MZRW and placebo groups were 43.3% and 8.3% during treatment, and 30.0% and 15.0% in the follow-up period, respectively (*P* <0.05). Those in the MZRW group showed benefits in terms of increased bowel movement, relief in the severity of constipation and straining, and effective reduction in the use of rescue therapy when compared with placebo. These encouraging results suggested that the higher dose, which had not been the recommended dosage in the Clinical Handbook of Chinese Herbal Formulae [13], was in fact safe and effective for alleviating FC when compared with the placebo [14].

In the current trial, we seek to evaluate the efficacy and safety of MZRW by comparing it with the commonly used stimulant laxative senna, as well as with a placebo, for patients with FC diagnosed with the TCM excessive syndrome. Senna is a stimulant laxative which facilitates the passage of stools by altering intestinal electrolyte transport and increasing intestinal motor activity [15]. Although no placebo-controlled trials were found, laxatives containing senna and fiber were more efficacious than lactulose [4]. Up to 26% of the patients underwent successful relief of impaction with senna combined with mineral oil [16]. Due to its low cost, safety and ease of ingestion, senna is widely adopted and prescribed in clinical practice. The use of senna remains the first-line treatment for the management of constipation according to the local guidelines of internal medicine published by the Hospital Authority, Hong Kong [17].

The study was financially supported by Food and Health Bureau, Hong Kong, China, through its Health and Health Services Research Fund (project no. 09101501) and was registered with an identifier (NCT01695850) in Clinical Trial.gov. This funding source had no role in the design of this study and will not have any responsibility during its execution, analyses, interpretation of the data, or decision to submit results.

## Materials and methods

### Study design

**Figure 1:**
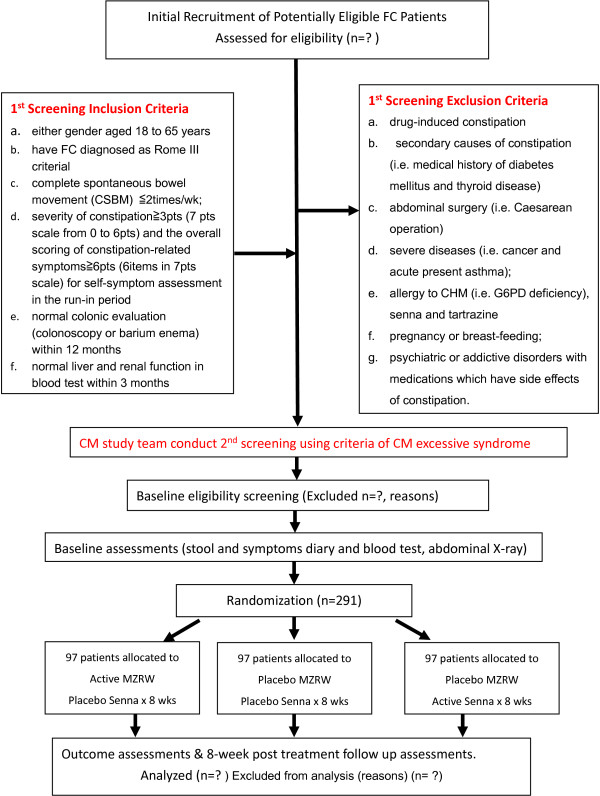
Participant flowchart.

**Figure 2:**
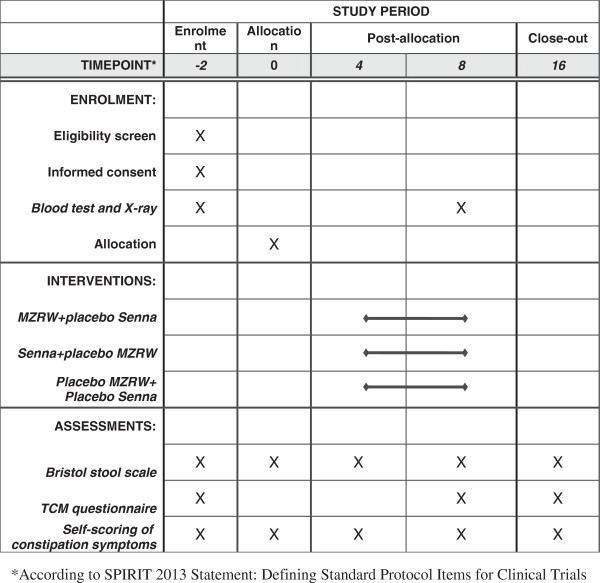
**Schedule of enrolment, intervention and assessments.**

This is a double-blinded, double-dummy, and placebo-controlled trial with three parallel groups. The protocol design was based on the recommendations made in the 'Design of Treatment Trial for Functional Gastrointestinal Disorders’, as proposed by the Rome III Working Team [3] and the Consolidated Standards of Reporting Trials (CONSORT) statement [18]. Two hundred and ninety-one participants were recruited from Lee Kee Memorial Dispensary (General outpatient clinic), Li Ka Shing Specialist Clinic of Prince of Wales Hospital and clinics of the School of Chinese Medicine, Hong Kong Baptist University. After a 2-week run-in period, eligible subjects were randomly assigned to one of three arms, the Chinese medicine (CHM) arm, the western medicine (WM) arm, and the placebo arm. The CHM arm received MZRW and WM placebo, the WM arm received senna and the CHM placebo, and the placebo arm received the CHM placebo and the WM placebo. All three groups then underwent an 8-week treatment and an 8-week follow-up period. Five visits in total were scheduled for each subject: one visit each in week 0, week 2, week 4, week 8 and week 16. The participant flowchart is listed in Figure 1 and the participant timeline is listed in Figure 2. The study protocol was approved by the Hong Kong Baptist University Ethics Committee on the Use of Human Subjects for Teaching and Research (Approval no. HASC/10-11/16) and the Joint Chinese University of Hong Kong-New Territories East Cluster Clinical Research Ethics Committee (Approval no. CRE-2013.192-T).

### Participants

*Setting*: The study was performed at Lee Kee Memorial Dispensary (general outpatient clinic), Li Ka Shing Specialist Clinic of Prince of Wales Hospital and Clinics of the School of Chinese Medicine, and Hong Kong Baptist University. The participants were enrolled from the public through advertisements in local newspaper and out-patient clinics.

*Diagnostic criteria for FC* (*Rome III*) [3]: 1) At least two of the following occurrences more than 25% of the time were required for a diagnosis of functional constipation: straining, lumpy or hard stools, sensation of incomplete evacuation, sensation of anorectal obstruction/blockage, manual maneuvers to facilitate, or fewer than three defecations per week; as well as 2) the rare presence of loose stools without the use of laxatives; and also 3) insufficient criteria for a diagnosis of irritable bowel syndrome.

*Diagnostic criteria for excessive constipation in TCM theory*[6]: Any three of the chief symptom manifestations of Heat and/or Qi stagnation were required for a diagnosis of functional constipation: 1) dry, hard stools; 2) difficult bowel movements; 3) abdominal distension, with or without tenderness; 4) belching; 5) dry mouth or halitosis; 6) red tongue with dry and/or yellow coating; and 7) Wiry pulse.

*Inclusion Criteria*: Patients were included if they had all of the following: 1) met the diagnostic criteria for FC (Rome III); 2) met the diagnostic criteria for excessive constipation in TCM theory; 3) were age 18 to 65 years (inclusive); 4) had a complete spontaneous bowel movement (CSBM) ≦2 times/week (CSBM is defined by feeling of complete passage of stool after defecation, rather than partial or incomplete evacuation, without the use of any laxative or enema within 24 hours) [19]; 5) exhibited severity of constipation ≧3 points (on a 7-point scale) [20]; 6) had a total symptom score ≧8 points (on a 7-point scale for constipation-related symptoms); 7) had a normal colonic examination (barium enema or colonoscopy) within five years; and 8) had a normal liver and renal function in blood test within 3 months.

*Exclusion criteria*: Patients were excluded if they had one or more of the following: 1) drug-induced constipation; 2) secondary causes of constipation (that is, medical history of diabetes mellitus, unstable thyroid disease); 3) abdominal surgery within one year (that is, Caesarean operation); 4) severe diseases (that is, cancer, acute asthma); 5) allergy to CHM (that is, G6PD deficiency); 6) pregnancy or breast-feeding; or 7) psychiatric or addictive disorders requiring medications with side effects of constipation.

### Interventions

*CHM intervention*: As reported previously, MZRW is composed of *Fructus Cannabis* (HuoMaRen), *Radix et Rhizoma Rhei* (DaHuang), *Radix Paeoniae Alba* (BaiShao), *Semen Armeniacae Amarum* (KuXingRen), *Fructus Aurantii Immaturus* (ZhiShi) and *Cortex Magnoliae Officinalis* (HouPo) [10]. The composition and action of each herb are summarized in Table 1[17]. The placebo was made from dextrin (76.03%), tea essence (23.61%), gardeniaflavin (0.02%) and *caramel (0.34%) to achieve color, smell, taste and texture comparable to MZRW granules. Patients were instructed to dissolve a sachet of granules (7.5 g) in 150 ml of hot water; and to* take the solution orally twice daily for 8 weeks. Quality control of the crude herbs authentication report is listed in Additional file 1.

**Table 1:** Composition and action of Ma Zi Ren Wan (MZRW)

**Ingredients**	**%**	**g/sachet**	**g/day**	**Action**
*Fructus Cannabis*	35.7	10.8^a^	21.7	TCM: Moistening the bowel and relieving constipation
				Pharmaceutical study: 1. Purgative effect, 2. Hypotensive effect
*Radix et Rhizoma Rhei*	17.9	5.4	10.9	TCM: Draining heat and relieving constipation
				Pharmaceutical study: 1. Purgative effect, 2. Antimicrobial effect, 3. Antineoplastic and ant mutagenic effect, 4. Hemostatic effect, 5. Immunosuppressive effect, 6. Choleretic effect
*Semen Armeniacae Amarum*	17.9	5.4	10.9	TCM: Reliving constipation by guiding Qi downward
				Pharmaceutical study: 1. Laxative effect, 2. Antitussive and antiasthmatic effect, 3. Analgesic effect, 4. Antineoplastic and antimutagenic effect
*Radix Paeoniae Albo*	8.9	2.7	5.4	TCM: Nourishing Yin and retaining homeostasis
				Pharmaceutical study: 1. Analgesic effect, 2. Antimicrobial and anti-inflammatory effect, 3. Immunologic effect
*Cortex Magnoliae Officinalis*	10.7	3.3	6.5	TCM: Promoting circulation of Qi and relieving flatulence
				Pharmaceutical study: 1. Central muscle-relaxation effect, 2. Anti-emetic effect, 3. Anti-ulcerative effect 4. Central inhibitory effect, 5. Antimicrobial effect
*Fructus Aurantll Immaturus*	8.9	2.7	5.4	TCM: Promoting circulation of Qi and relieving flatulence
				Pharmaceutical study: 1. Diuretic effect, 2. Two-way effect on gastrointestinal smooth muscle, 3. Cardiovascular stimulating effect

*TCM*, traditional Chinese medicine.

^a^Each gram of *MZRW* granule is equivalent to 4.05 g of raw herbs.

*WM intervention*: An over-the-counter drug, Senokot, was selected as the active control. Each tablet comprised 7.5 mg of Sennodide B [21]. Tablets were manufactured by Reckitt Benckiser Company as circular, biconvex, greenish brown tablets. The placebo was made of starch and color to achieve an appearance comparable to Senokot. Patients were instructed to take 2 tablets at bedtime for 8 weeks. The Senokot placebo tablet was made by Guangzhou Hua hai Pharmaceuticals Co., Ltd,China according to the standards of Good Manufactory Practice (GMP).

*Rescue medication*/*enema*: Dulcolax tablets and Dulcolax suppositories [22] were provided to ensure bowel movements only in those patients without bowel movements for at least three consecutive days during the study. The rescue medication was manufactured by Boehringer Ingelheim Pharmaceuticals, Inc.,U.S.A.

MZRW granules and placebos were prepared by PuraPharm (Nanning) Pharmaceuticals Col, Ltd.,China. (lot nos. A1201839 for MZRW, and A1202071 for placebos, respectively). The entire manufacturing process, from authenticating the raw materials to the final product, was in strict compliance with the standards of Good Manufactory Practice (GMP) and Chinese Pharmacopoeia 2010 [23]. Acute toxicity testing of MZRW granules was performed on mice, in order to monitor for toxicity of the formula. The results showed that the maximum tolerance dosage was 75 g/kg, equal to 300 times the dose tested (Table 2). Chemical composition of the final products were analyzed for contamination with heavy metals, toxic elements, microbes and pesticide residue. Both MZRW and CHM placebo granules were packed in sealed opaque aluminium sachets and put in zip lock bags (28 sachets each) while Senokot and WM placebo were put in plastic bottles (56 tablets each). Only the treatment code was printed outside the package to ensure successful blinding of patients.

**Table 2:** **Acute**-**toxicity of Ma Zi Ren Wan (MZRW)**

			**Dosage g/kg**		
	**75**	**37.5**	**19.25**	**9.625**	**4.8125**
Before administered drug	22.2 ± 1.1	20.4 ± 0.6	21.1 ± 1.6	21.8 ± 1.7	23.3 ± 1.9
After administered drug	28.7 ± 4.1	25.5 ± 3.0	25.5 ± 1.1	26.1 ± 1.1	30.3 ± 1.6

Sixty ICR mice, which were divided into five groups, were used in the acute-toxic test. The mice weighed 18 to 22 g. The drug was dissolved with distilled water and administered 20 ml/kg orally. The details about dosage were as follows: 75 g/kg, 37.5 g/kg, 19.25 g/kg, 9.625 g/kg, and 4.8125 g/kg. The dosages equaled 300, 150, 75, 37.5, and 19.75 times of clinical dosage. Untoward reaction did not be observed after mice were administered the drug. Administered drug 7 days later, no one died. The weight of mice was listed as Table 2.

### Outcomes

Participants recorded stool frequency, stool form (on the 7-point Bristol stool scale, ranging from 'Separate hard lumps’ to 'Watery’), and feeling of complete evacuation (yes/no). They were also required to log intake of research medication, rescue drug and/or any other medication use throughout the study in a daily diary. Principal investigators (PIs) and research assistants (RAs) interviewed patients at the end of Week 2 (baseline), Week 4 (within treatment), Week 8 (end of treatment), and Week 16 (end of follow-up), for symptoms, compliance, and occurrence of adverse events. Data on global symptom improvement, individual bowel symptoms and stool consistency were collected.

Individual assessment of constipation and related symptoms (severity of constipation, sensation of straining, incomplete evacuation, bloating, abdominal pain/cramping, nausea and passing of gas) were recorded using a 7-point ordinal scale (0 = not at all, 6 = very severe) [see Additional file 2: Table S1] on a designated form that participants filled out during every visit [3]. Global symptom improvement was defined as the participant's subjective feeling of adequate relief of symptoms. For this purpose, participants were asked to rate their impression of changes in constipation by scoring (in comparison with their baseline (Week 2) scores) their impression of these changes from 0 to 6 to represent that they were feeling markedly worse or better at the respective extremes [3]. The response categories are collapsed to simply 'Improved’ for score 4 to 6, 'Same’ for score 3 or 'Worse’ for score 0 to 2 [24].

The primary outcome will be the response rate for CSBM during the treatment period: a clinically meaningful endpoint that combines an objective measure (number of bowel movement) with a subjective measure (feelings of patients as to completeness of defecation) [25,26]. Participants with a mean increase of CSBM ≧1/week compared with the last 14 days of the run-in period are defined as responders.

Secondary outcomes will include the response rate for CSBM during the follow-up period, including stool frequency, changes from baseline in scores of individual symptom assessment (severity of constipation, sensation of straining, incomplete evacuation, bloating, cramping/abdominal pain, nausea and passing of gas), and global symptom assessment (improved, same or worse). Safety profiles of MZRW will be assessed by determining important adverse events reported in participants’ diaries, in follow-up interviews and in clinical laboratory evaluations (for example, liver and renal function testing). During the last visit, the success of blinding would be evaluated for both investigators and patients, as to whether CHM, WM or placebo had been taken. In particular, patients are asked to rate the appearance, color, texture, taste and efficacy of the medication to try to determine whether these factors were important in their conclusion.

In addition, an objective estimate of changes in colonic transit time is made based on a commercially available radio-opaque Sitzmarks capsule (Konsyl Pharmaceuticals, INC.,Easton, MD 21601,U.S.A).

Each gelatin capsule contains 24 barium sulfate embedded polyvinyl chloride markers measuring 1 mm × 4.5 mm. Plain radiographs of the abdomen are obtained after patients swallow a capsule, for five days (120 hours) before and 8 weeks after the treatment period. Normal colonic transit is defined as expulsion of at least 80% (19 or more) markers [27,28]. For patients with six or more makers scattered throughout the colon, hypomotility or colonic inertia is suggested. If six or more markers are gathered in the rectosigmoid region with a near normal transit of markers through the rest of the colon, functional obstructive or dys-synergic defecation is suspected [4,29].

### Recruitment procedures

Three strategies are used to recruit participants with FC. The first relies on the participant bank of over 600 patients, built up from prior research team work in functional constipation related studies since 2007. The second relies on referrals from the two study sites (Lee Kee Memorial Dispensary and Li Ka Shing Specialist Clinic of Prince of Wales Hospital) from our co-investigators (Co-Is). The third strategy consists of posted advertisement in local newspapers and a press conference to introduce our research.

All patients diagnosed with functional constipation by the physicians (Co-Is) are referred to the principal investigator (PI) and/or to the research assistants (RA) to determine whether they meet diagnostic criteria of excessive constipation according to TCM theory. There is inconsistency among different TCM practitioners regarding pattern differentiation for FC. If any inconsistency occurs for an individual patient concerning pattern identification, the other senior CMP with more than 20 years clinical practice will be invited to make the diagnosis of TCM pattern independently to ensure the consistence of the diagnosis. The aims, procedures and nature of the study, and possible side effects are explained by the PI and/or the RAs before a written consent form is obtained from each subject who agrees to participate. Moreover, patients are informed that they are free to withdraw at any time during the study. All patients undergo a 2-week, drug-free observation period. Meanwhile, they have to complete a stool and symptom diary to confirm their diagnosis. Blood tests of liver and renal function (blood urea, creatinine, alanine transaminase and aspartate transaminase, alkaline phosphatase and bilirubin), and plain abdominal X-ray for colonic transit study are arranged. Patients are considered eligible for the study if their diaries reflect conformance with the Rome III criteria for a diagnosis of functional constipation, and if they also have normal liver and renal function. Recruited patients will obtain their intervention assignment from the PI and/or RAs.

### Assignment and blinding

Block randomization was carried out in 1:1:1 ratio according to the sequence generated with Random Allocation Software (Version 1.0.0), Isfahan, Iran. The RA assigns treatments according to the codes that are kept in opaque sealed envelopes with consecutive randomization numbers. Treatment assignments will not be revealed and are blinded to the patients and investigators (including statisticians) until the entire study is completed. At the last visit, all patients and the PI/RAs will complete a questionnaire about which treatment (CHM group, WM group or placebo group) the patients received to evaluate the success of blinding. Code breaks should only occur in exceptional circumstances when knowledge of the actual treatment is absolutely essential for further management of the patient.

### Sample size calculation

From the results of the placebo-control study, the response rate of MZRW was 43.3% [14]. Assuming the response rates for TCM, senna and placebo were 40%, 20% and 10%, respectively. Therefore, 82 patients per treatment group were deemed sufficient to achieve 80% power in detecting treatment differences, based on two-sided Chi-square test without continuity correction at a significance level of 0.025 (used to maintain the overall significance level at 5%). Further, assuming a 15% dropout rate, we concluded that a total of 291 patients (97 per arm) would need to be recruited to ensure statistically significant results. The calculation is performed using StudySize 2.0 software, London, UK.

### Statistical analysis

All efficacy and safety analyses will be conducted according to the intention-to-treat (ITT) principle. Missing values will be imputed by the last-observation-carried-forward method. The statistical analysis will be performed using the Statistical Packages of Social Sciences (SPSS) for Windows version 16.0. The statistical significance is defined as two-sided *P* value of <0.05. Baseline characteristics will be reported as mean (SD). Baseline differences among the groups will be assessed with the use of Student’s t-test for normally distributed continuous variables and the non-parametric Mann-Whitney U test for non-normally distributed variables. For categorical variables, chi-squared test or Fisher’s exact test will be used. Comparisons between groups will be conducted by using an analysis of covariance (ANCOVA) with baseline as covariate. All items and subscales will be compared between groups for each 6-week treatment using ANCOVA, with treatment group as a factor in the model and baseline as the covariate. The changes from baseline to endpoint of treatment in scores will be tested with repeated measure analysis of variance (ANOVA). Within group differences will be assessed with paired t-test for normally distributed data and Wilcoxon signed-rank test for non-normally distributed data.

### Data collection and handling of withdraw and dropout

This is an 18 week clinical trial, in which subjects need to take research medication for 8 weeks, attend 5 assessment visits, obtain 2 sets of blood tests and plain abdominal X-rays, fill in a few sets of questionnaires, complete a patient diary for the entire study period, and stop taking other herbal medication and laxatives. Original data of study forms will be entered and kept on file at the participating site. All the files are to be stored in numerical order and stored in a secure and accessible place. Participant files will be maintained in storage for a period of 5 years.

In order to maximize subjects’ compliance, we first run a thorough consent process for all participants by explaining the details of the study schedule, potential side effects of treatment, and the responsibilities that need to be undertaken by subjects. Second, support and reassurance is provided during the whole study. Third, we will carefully scrutinize subjects (during the 2-week run-in period) to exclude ineligible and potentially low compliance individuals before randomization. Fourth, a direct telephone hotline and email account were established in order to optimize active communication with patients and to respond to enquiries. Furthermore, additional visits can be arranged for patients to see WM doctors or TCM practitioners if patients develop adverse events before the next scheduled visit. If any patient expressed thoughts of withdrawing or dropping out, we would try to help that individual determine the reason, and attempt to resolve any issues, in order to keep the patient in the study.

In order to monitor the reported compliance of the patients, the patients will be required to return the remaining medications at treatment visits except the first visit and last visit. Our research assistant will take a record of each patient. 80% of the consumption will be considered good compliance. If the patients have no bowel movement for at least three consecutive days during the study rescue therapy as dulcolax suppository are provided to the patients.

The trial would be terminated in a specific subject if he/she has: 1) presence of severe adverse effect; 2) hypersensitivity towards research medication; 3) participation in another CHM research project; or 4) presence of life-threatening disease. The whole research plan would be terminated for the following circumstances: 1) presence of serious adverse effect related to the research medication with supportive evidence; or 2) completion of all follow-up assessments.

## Discussion

This randomized double-blind clinical trial is the third clinical study our team has conducted on the effectiveness of MZRW in FC patients with 'excessive syndrome’ in TCM diagnosis. The first study was the optimal dose determination study comparing three different dose groups [13]. The second trial was to evaluate the effectiveness of the optimal dose determined in the previous study compared to the placebo [13]. To our knowledge, this study is the first study to compare the Chinese herbal proprietary medicine (MZRW) with commonly used laxative in the clinical practice of Western medicine, as well as placebo. This study will complete the investigation of MZRW, for the common condition FC, and seeks to provide supportive data to assist in informing clinical practice. Furthermore, we feel that this sequential approach can be replicated for determining clinical effectiveness of CHM interventions in the future. That is, the sequence comprising first conducting a systematic review [12], then implementing a dose determination study which is followed by a placebo-control trial, and lastly comparing with an active control in blinded clinical trial.

Another key process of the protocol is the use of combined diagnostic criteria for both the western diagnosis of Functional Constipation and the Chinese medicine diagnosis of excessive syndrome. In TCM, 'syndromes’ are the foundation for therapeutic principle. The identification of TCM syndromes in clinical practice is subjective and is highly dependent on personal experience, and thus that is why randomized clinical trials of TCM are often lack of syndrome diagnosis. However, by excluding TCM diagnoses, CHM studies must therefore also lose the most important characteristics of Chinese medicine treatment. In this study, we conduct the trial according to the logistical screening steps both in the specialist clinics of gastroenterology and clinical centre of School of Chinese Medicine in order to retain the optimal Chinese Medicine therapeutic effect. The CM diagnostic criteria were arrived at by consensus of all investigators prior to initiating the trial, and are based on the standard criteria described in *Internal Chinese Medicine* and *Criteria of Diagnosis and Therapeutic Effect of Disease and Syndromes in Traditional Chinese Medicine*.

Our systematic review of Chinese herbal medicine for functional constipation, which compared CHM with placebo/no treatment, conventional western medicine (WM), other CHM, and non-pharmaceutical interventions, also indicated the need to seek strong evidence from well-designed CHM clinical trials. The review analyzed 35 randomized trials of CHM interventions and favored the effectiveness of CHM in comparison with various controls [12]. However, it is difficult to translate the results of such studies due to the low methodological quality, different compositions of the CHM formula and lack of standard dosage preparations or manufacturing processes. Strengths of this trial include our rigorous methodology and strict quality control interventions. We described our method of recruitment, sequence generation, allocation concealment mechanism, and data collection methods in detail. In addition, we reported the CHM intervention, MZRW according to *Recommendations for Reporting Randomized Controlled Trials of Herbal Interventions*[30], including dosage regimen and quantitative description, the indications of CM theory, the standardization of the products, and so on.

The clinical trial protocol serves as the foundation, playing a key role of study planning conduct reporting and appraisal. To facilitate appropriate reference standards for scientific, ethical and safety issues before the trial begins, this protocol has been developed according to Consolidated Standards of Reporting Trials (CONSORT) statement [18], Standard Protocol Items: Recommendations for Interventional Trials (SPIRIT) 2013 [31] and SPIRIT 2013 explanation and elaboration: guidance for protocols of clinical trials [32].

## Conclusions

In conclusion, the results of this study are expected to provide further consolidated evidence for the safety and effectiveness of the CHM patent medication, MZRW, for treatment of functional constipation in patients who also meet diagnostic criteria for a TCM pattern of Excessive syndrome.

## Trial status

At the time of manuscript submission, the study has been actively enrolling participants for one month. During this enrollment period, a total of 20 subjects have been enrolled; no one has completed the treatment yet.

## Abbreviations

ANCOVA: Analysis of covariance; ANOVA: Analysis of variance; CHM: Chinese herbal medicine; Co-I: Co-investigator; CONSORT: Consolidated standards of reporting trials; CSBM: Complete spontaneous bowel movement; FC: Functional constipation; G6PD: Glucose-6-phosphate dehydrogenase deficiency; HD: Higher dose; ITT: Intention-to-treat; MZRW: Ma Zi Ren Wan; PI: Principal investigator; RA: Research assistant; SD: Standard deviation; SPIRIT: Standard protocol items: recommendations for interventional trials; SPSS: The statistical packages of social sciences; TCM: Traditional Chinese medicine; WM: Western medicine.

## Competing interests

The authors guarantee that there exists no competing interest in this paper. Neither the funding agency nor any outside organization had a role in study design or manuscript preparation. The authors declare that they have no competing interests.

## Authors’ contributions

All authors participated in the design of the study and performed the trial. LZ drafted the manuscript. JW and ZB supervised and coordinated the clinical trial. YC participated in general trial coordination. KC, TL and XC referred and recruited patients. CC participated in randomization and statistical analysis. CTW is responsible for recruiting the participants. All authors read and approved the final manuscript.

## Supplementary Material

Additional file 1 **Crude herbs authentication report for the efficacy and safety study of MZRW.** The contents determination of *Cortex Magnoliae Officinalis* and *Radix et Rhizoma Rhei* in MZRW were carried out by using high-performance liquid chromatography (HPLC) and the results are listed in Additional file 1. Click here for file

Additional file 2: Table S1 Individual Assessment of Constipation and Related Symptoms. Click here for file
