# Supplementary material for: Chinese herbal medicine (Ma Zi Ren Wan) for functional constipation: study protocol for a prospective, double-blinded, double-dummy, randomized controlled trial
Source: Trials. 2013 Nov 4;14:366. doi: 10.1186/1745-6215-14-366 (PMC4228394; doi:10.1186/1745-6215-14-366)
Supplement: Additional file 1 — Crude herbs authentication report for the efficacy and safety study of MZRW. The contents determination of Cortex Magnoliae Officinalis and Radix et Rhizoma Rhei in MZRW were carried out by using high-performance liquid chromatography (HPLC) and the results are listed in Additional file 1. [file 1745-6215-14-366-S1.docx]

**Additional file 1: Crude herbs authentication report for the efficacy and safety study of MZRW**

The contents determination of *Cortex Magnoliae Officinalis* and *Radix et Rhizoma Rhei* in MZRW were carried out by using high-performance liquid chromatography (HPLC) based on The Chinese pharmacopoeia (the second edition). The results are below.

| Herb | Testing index | Result (%，g/g) | Content limitation | The appendix |
| --- | --- | --- | --- | --- |
| *Cortex Magnoliae Officinalis* | Magnolol + honokiol | 3.1 | ≥2.0% | 1 |
| *Radix et Rhizoma Rhei* | Aloe-emodin, rhein, emodin, Chrysophanol, Physcion | 1.5 | ≥1.5%  (Total contents) | 2 |

**(1) HPLC detection of Contents of magnolol and honokiol from *Cortex Magnoliae Officinalis* (The Chinese pharmacopoeia, 2005)**

**Crude sample preparation:**

The powder (0.2g) was added to 25 mL of methanol in the sealed conical flask. The mixture was shaken well and immersed for 24 hours. The 5 ml of filtered supernatants were added to 25 mL of methanol and shaken. The extraction was analyzed by using HPLC.

**Control sample:** The concentration of magnolol – methanol solvent was 40.0µg/mL. The concentration of honokiol – methanol solvent was 20.0µg/mL.

**HPLC analysis:** The column used was a Ultrasphere C18 column. The mobile phase was methanol-water (78/22, v/v). The effluent was monitored at 294 nm. The loading amount of sample was 5 µL.

**Chromatograms of magnolol and honokiol by HPLC:**

Attached 10: Fig1-1. Chromatograms of control samples of magnolol and honokiol by HPLC.

Attached 10: Fig1-2. Chromatograms of crude sample 1 of magnolol by HPLC.

Attached 10: Fig1-3. Chromatograms of crude sample 2 of magnolol by HPLC.

**Results：**

|  | Sample size  (g) | Magnolol | | Konokiol | | Total contents |
| --- | --- | --- | --- | --- | --- | --- |
|  |  | Peak area (mAU) | Contents  (g/g, %) | Peak area (mAU) | Contents  (g/g, %) |  |
| Control sample |  | 309.9 | 100% | 169.1 | 100% |  |
| Crude sample 1 | 0.2025 | 259.5 | 2.07 | 153.5 | 1.12 | 3.2 |
| Crude sample2 | 0.2012 | 248.5 | 1.99 | 147.0 | 1.08 | 3.1 |
| Average |  |  |  |  |  | 3.1 |

**(2) HPLC detection of Contents of anthraquinones compounds from *Radix et Rhizoma Rhei* (The Chinese pharmacopoeia, 2005)**

**Crude sample preparation:**

The powder (0.15g) sifted at No.4 seive was added to 25 mL of methanol in the sealed conical flask. The mixture was weighed well and was heated to reflux for 1 hour. Methanol was added to reach the initial weight of the mixture after cooled down to room temperature. It is then shaken and filtered. The 5 ml of filtered supernatants were added with 10ml of Hydrochloric Acid (8%). The mixture was added with 10ml of chloroform after treated with ultrasonic for 2 minutes. It was then heated to reflux for 1 hour and was cooled down to room temperature. The organic phase was separated with a separatory funnel. The acid solution was extracted with 10 ml chloroform three times. The chloroform solution were combined and dried by vacuum concentration. The remains was added to 10 ml of methanol. The mixture was shaken well and filtered. The filtered supernatants were saved.

**Control samples:** The concentration of Aloe-emodin, rhein, emodin and Chrysophanol were 16µg/mL, respectively. The concentration of Physcion was 8µg/mL.

**HPLCanalysis:** The column used was an Ultrasphere C18 column. The mobile phase was methanol-0.1% **phosphorous acid** (85/15, v/v). The effluent was monitored at 254 nm. The loading amount of sample was 10 µL.

**Chromatograms of Aloe-emodin, rhein, emodin, Chrysophanol and Physcion by HPLC:**

Attached 10: Fig2-1. Chromatograms of control samples of Aloe-emodin (1), rhein (2), emodin (3), Chrysophanol (4) and Physcion (5) by HPLC.

Attached 10: Fig2-2. Chromatograms of crude sample 1 of Aloe-emodin (1), rhein (2), emodin (3), Chrysophanol (4) and Physcion (5) by HPLC.

Attached 10: Fig2-3. Chromatograms of crude sample 2 of Aloe-emodin (1), rhein (2), emodin (3), Chrysophanol (4) and Physcion (5) by HPLC.

**Results：**

|  |  | Control sample | Crude sample 1 | Crude sample 2 |
| --- | --- | --- | --- | --- |
| Aloe-emodin | Peak area (mAU) | 523.0 | 336.2 | 270.4 |
|  | Contents  (mg) |  | 0.514 | 0.414 |
| rhein | Peak area (mAU) | 718.9 | 182.8 | 272.9 |
|  | Contents  (mg) |  | 0.203 | 0.304 |
| emodin | Peak area (mAU) | 795.9 | 305.4 | 300.2 |
|  | Contents  (mg) |  | 0.307 | 0.302 |
| Chrysophanol | Peak area (mAU) | 778.4 | 912.5 | 869.4 |
|  | Contents  (mg) |  | 0.938 | 0.894 |
| Physcion | Peak area (mAU) | 224.5 | 176.0 | 171.3 |
|  | Contents  (mg) |  | 0.314 | 0.305 |
| Total amounts (mg) |  |  | 2.276 | 2.218 |
| Sample weight (mg) |  |  | 150.1 | 151.1 |
| Contents (%) |  |  | 1.52 | 1.47 |
| Average (%) |  |  | 1.5 | |
