# Supplementary material for: Chinese herbal medicine (Ma Zi Ren Wan) for functional constipation: study protocol for a prospective, double-blinded, double-dummy, randomized controlled trial
Source: Trials. 2013 Nov 4;14:366. doi: 10.1186/1745-6215-14-366 (PMC4228394; doi:10.1186/1745-6215-14-366)
Supplement: Additional file 2: Table S1 — Individual Assessment of Constipation and Related Symptoms. [file 1745-6215-14-366-S2.doc]

**Additional file 2: Table S1** Individual Assessment of Constipation and Related Symptoms

| In the last seven days, please rate the severity of constipation and related symptoms:  0 ----------------------------------------------- Not at all  1 ----------------------------------------------- Minor  2 ----------------------------------------------- Mild  3 ----------------------------------------------- Moderate  4 ----------------------------------------------- Moderately severe  5 ----------------------------------------------- Severe  6 ----------------------------------------------- Very severe | | | | | | | |
| --- | --- | --- | --- | --- | --- | --- | --- |
| In the last seven day, have you ever experienced the symptoms listed as below? If any, please rate the severity of the symptoms. | | | | | | | |
|  | Not at all | Minor | Mild | Moderate | Moderate  severe | Severe | Very severe |
|  | | | | | | |
| Sensation of straining | 0 | 1 | 2 | 3 | 4 | 5 | 6 |
| Incomplete of evacuation | 0 | 1 | 2 | 3 | 4 | 5 | 6 |
| Bloating | 0 | 1 | 2 | 3 | 4 | 5 | 6 |
| Abdominal pain | 0 | 1 | 2 | 3 | 4 | 5 | 6 |
| Nausea | 0 | 1 | 2 | 3 | 4 | 5 | 6 |
| Passing of gas | 0 | 1 | 2 | 3 | 4 | 5 | 6 |
